# A New Processing Method for Laser Sintering Polymer Powders at Low Bed Temperatures

**DOI:** 10.3390/polym16233301

**Published:** 2024-11-26

**Authors:** Lanti Yang, Hao Gu, Zahir Bashir

**Affiliations:** 1 SABIC Analytical Science Europe, Corporate T&I, Plasticslaan 1, 4612 PX Bergen op Zoom, The Netherlands; 2 SABIC Global Application Technology Europe, Specialties, Plasticslaan 1, 4612 PX Bergen op Zoom, The Netherlands; hao.gu@sabic.com; 3 Catenated Carbon Consultancy Ltd., 192 Wake Green Road, Birmingham B13 9QE, UK; zbashir2703@gmail.com

**Keywords:** additive manufacturing, low bed temperature, laser sintering, powder, PET, PA12

## Abstract

Most current laser sintering (LS) machines for polymer powders operate with a maximum bed temperature of 200 °C, limiting the use of higher melting polymers like polyethylene terephthalate (PET), which melts at ~250 °C. Using bed temperatures of ≤200 °C leads to severe part-distortion due to curl and warpage during the sintering process. The paper presents a processing method for LS at low bed temperatures, using an in situ printed anchor film to conquer curl and warpage. With the use of the anchor film, PET parts were successfully printed without machine stoppage at bed temperatures as low as 150 °C, which is about 80 °C lower than the bed temperature for a regular process for PET without the anchor film. The anchor film acts as a frictional restraint, effectively preventing the curling and warping during printing that typically result from crystallization-induced shrinkage at low bed temperatures. Whereas previous studies have employed 13 mm thick anchoring sheets bolted to the machine to prevent curl and warpage at low bed temperatures, our method uses a flexible in situ printed ~70 μm thick film to which the built part naturally adheres. The in situ printed film is easily detachable from the part after the build. The standard LS material, polyamide 12 (PA12), was also printed with lowered bed temperaturewhere the benefit would be reduced thermal degradation of the powder and decreased energy consumption during the sintering process.

## 1. Introduction

Additive Manufacturing (AM) relies on building shaped articles through a layer-by-layer building process [1,2,3]. It has transitioned from a prototyping method to a widely adopted industrial production technique for both polymeric and non-polymeric materials.

Powder-bed fusion methods like laser sintering (LS) [4,5] and high-speed sintering (HSS) [6,7] utilize polymer powders as feedstock to fabricate objects. To enable the LS of semi-crystalline polymer powders, the materials are maintained in the powder bed at temperatures between the crystallization temperature (T_c_) and the melting temperature (T_m_) [8], typically 5–25 °C below the T_m_, to minimize curling during the sintering process [9]. Curling refers to the phenomenon that causes the upward bending of the part being built, resulting in protrusion over the powder bed. This can disrupt the positioning of the part, particularly when the applicator lays down the subsequent powder layer. Curl will be more prominent with semi-crystalline polymers due to the density different between the melt and crystalline phases, leading to significant shrinkage on solidification. Curl arises from variation in shrinkage rates due to temperature differences between the previous layer of melt, which has started to crystallize and shrink, and the newly melted material on top, which has shrunk less. The greater the temperature difference between the powder bed and the T_m_, the more pronounced the curling effect will be. Note that the effect of crystallization shrinkage in injection molding is controlled by the application of a “mold packing pressure”. However, in the powder-bed fusion process, such packing pressure cannot be utilized, making the control of curl more challenging.

Recently, we demonstrated that the powder of the more common polymer poly(ethylene terephthalate) (PET) is a suitable material for LS [10,11,12] and HSS [13]. As PET has a melting temperature of ~240–250 °C, the powder in the building area must be kept preheated at a temperature of 225–233 °C for sintering with limited curl and warpage of the parts. We had access to a special high-bed-temperature machine that allowed setting the optimum bed temperature of 225–233 °C for PET [10]. We showed HSS with PET powder was also possible but after some modification to the machine, whose maximum bed temperature was 200 °C [13]. The most commonly available LS and all HSS machines in industrial printing companies (called “service bureaus”) are currently limited to a maximum bed temperature of 200 °C. Consequently, these machines can only sinter semi-crystalline polymers with melting points below approximately 200–220 °C (PA12, PA11, polypropylene, and thermoplastic urethanes). This limitation presents a challenge for commercial use of PET and other high-temperature polymers such as polyphenylene sulfide (PPS) and polyether ether ketone (PEEK).

The major reason in the literature to seek methods to sinter with beds at low temperatures is to reduce the degradation of the unsintered (but heat-exposed) powder left in the bed after a build [14,15,16]. The reuse of the heat-exposed but unsintered powders in LS can result in defective articles [17]. As a common practice, manufacturers typically refresh the powder by adding a percentage of virgin material, such as mixing 50% virgin PA12 powder with heat-exposed PA12 powder [16]. Keeping the bed with polymer powder at elevated temperatures for hours leads to high energy consumption, hence lower bed temperatures would be beneficial. The primary motivation for this research was to laser sinter PET with a bed temperature <200 °C. This approach aims to make PET compatible with the low-temperature LS and HSS machines designed for PA12, which are widely available in commercial manufacturing.

There have been two categories of solutions explored for low bed temperature sintering: (1) use a “support structure” [18,19,20], which mounts to a thick rigid plate (typically >10 mm in thickness) to anchor the part being built, and thereby control shrinkage and curl (2) optimize the energy density and laser scanning pattern [21,22] without the need for building a “support structure”.

In the literature, the support structure is typically represented as a rigid, thick sheet (approximately 13 mm) made from a material that is similar or compatible with the one being laser sintered. Building the support structure often involves a complex fabrication process, which may include high-temperature LS or injection molding [18,19,20]. For example, Niino et al. [18] developed an LS process that represents an advancement in low bed-temperature sintering by eliminating the need for powder-bed preheating. In their study, both PA12 and the biopolymer poly(glycolic acid) were laser sintered with the powder bed maintained at ambient temperature. Since high curl is anticipated under these conditions, a 13 mm thick base plate of the polymer being sintered was fabricated using conventional (high-bed-temperature) LS and subsequently bolted to an aluminum plate to help control this issue for PA12. The ease of detachment of the parts from this rigid support was not disclosed, and it is expected that the efficiency of the LS process may be reduced as a result. Menge and Schmid [20] explored low bed temperature LS of PA12 (80–120 °C) to reduce powder degradation. In their method, a building platform must be engineered out of a fiber composite material whose matrix polymer, after melting, would be compatible with the melt of the material from which the part is made. The thickness of the building platform, the method of its fabrication (injection molding or extrusion), and the ease of debonding the parts from the building platform were not disclosed. There is a method developed by a consortium of an aerospace company, a LS machine manufacturer and a powder supplier where polyether ether ketone (PEEK) powder with T_m_ of ~ 340 °C was printed with low bed temperature using a rigid support (see Figure 1 in Ref. [20]). The powder was laid over a metal plate having a grid of wells (~ 3 mm x 3 mm, 4 mm deep) and a ~4 mm thick PEEK support sheet was laser sintered over it. Thin struts or legs were printed on the PEEK sheet and the PEEK article were printed on the legs, which can be broken off. Designing and printing of the legs was cumbersome. For low-temperature printing avoiding support structures, Schlicht et al. [22] explored a low powder-bed fusion method using “Fractal Quasi-Simultaneous Exposure Strategies” for PA12. This approach utilized low bed temperatures and quasi-simultaneous fractal scan paths for the laser. The quasi-simultaneous scanning increases layer scanning time due to the repetitive exposure of distinct segments, in contrast to the traditional single exposure by the laser beam. Though curl and warpage could be avoided without support structures for LS of PA12 with low bed temperatures of 75 °C and 100 °C, the sintered parts showed a layered morphology. A more complex part, such as a PA12 spring, demonstrated roughness and geometric inaccuracy [22]. In all these published methods (support structure or adjusting laser energy input), compromises must be made regarding part quality and production speed.

In this work, a much simpler anchoring method was explored to laser sinter parts from PET with lowered building-area temperature, enabling the possibility to use PET in LS machines commonly found in today’s industrial service bureaus. Unlike previous methods that employed rigid supports, this approach utilizes flexible support created in situ from the same polymer being processed, eliminating the need for bolting to the machine and allowing for easy detachment from the final part. This low-temperature LS method was also demonstrated to be effective for PA12, facilitating the production of an object with a complex shape.

## 2. Materials and Methods

### 2.1. PET and PA12 Powder

A semi-crystalline PET powder (SABIC, Yanbu, Saudi Arabia) with an intrinsic viscosity of 1.122 dL/g was used. The crystallinity measured by Differential Scanning Calorimeter (DSC) was about 50%. The particle size distribution (PSD) was measured via a Malvern Mastersizer 2000 (Malvern Panalytical, Almelo, The Netherlands) with the results of 23 µm for D10, 46 µm for D50, and 89 µm for D90 [11]. To demonstrate the potential application of the method to other polymer materials for LS, Duraform^®^ PA12 (from 3D Systems, Rock Hill, USA.) was also selected in this study [23].

### 2.2. LS of PET at Low Bed Temperature

#### 2.2.1. LS of PET Under “Standard Conditions”

A laboratory type LS machine was used for printing PET in this study. This machine could, in fact, reach bed temperatures up to 250 °C, but here it was used at temperatures below 250 °C. The LS process for PET must be conducted in a dry nitrogen atmosphere with an oxygen content of ≤1.0 wt.%. This is essential to avoid molecular weight drop and yellowing/browning of the PET. The same equipment had been used in our previous work involving LS of pure PET powder [10,11,12] and an aluminum-PET mixed powder [24]. For PET, we established a ‘standard’ build-bed temperature of ~225–233 °C (about 20 °C below the onset of melting) was needed to avoid curl in the parts. Thus, ~225–233 °C can be considered to be the optimum bed temperature for a “standard process” (that is, without anchoring support) for PET. Printing was first tested by laser sintering tensile bars to duplicate previous results. The previous findings were found to be repeatable. To test the ability of the LS process at a lower bed temperature than standard conditions, we also attempted to print the tensile bars and rectangular bars with the bed temperature for the PET lowered to 210 °C. The curl was so severe that continued printing was not possible due to machine stoppage. A temperature of 210 °C is about 40 °C lower than the melting peak of PET, and with such a low bed temperature, curl aborts the printing process.

#### 2.2.2. LS of PET at Low Bed Temperature with an In Situ Produced Anchor Film

**Figure 1:**
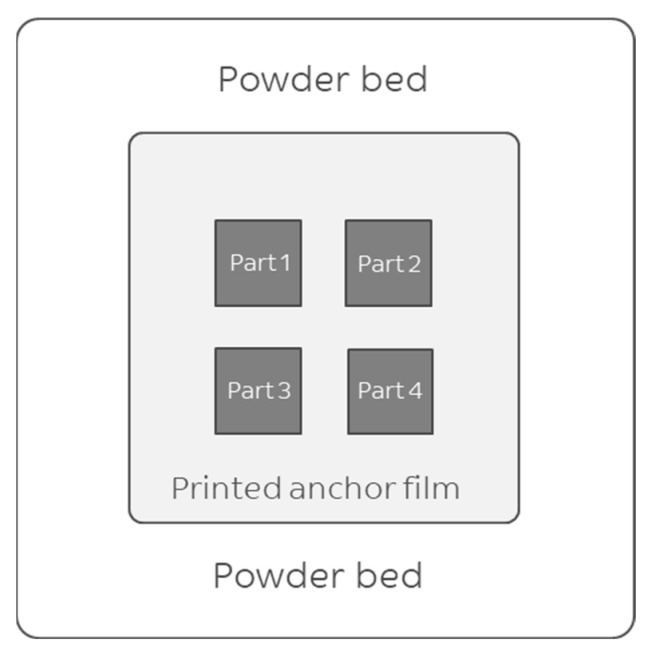
The print layout in the experiments to test the feasibility of printing PET at six low bed temperatures. A 10 cm × 10 cm anchor film was printed first. Then, 4 square plates were printed on the film, each at a different power setting.

Square plates were selected over tensile bars for this study, as they allow for rapid assessment of common LS defects, such as curling, mis-shaped corners, and delamination. Flat plates with sharp edges and corners, as well as high density, indicate that more complex shapes will also print successfully. The following method was developed to enable the LS of square plates at low bed temperatures. As shown in Figure 1, a thin anchor film 10 cm × 10 cm was printed first. After this step, four square plates (30 mm × 30 mm) were then printed over the thin film. The LS process settings for printing the PET anchor film and the square plates are summarized in Table 1 for each experiment. Eight experiments (from A1 to A8) with various building-area temperature (°C) and laser powers were conducted. The bed temperature was lowered in each experiment (200, 190, 180, 170, 160, and 150 °C) while three processing settings were set to fixed values: scan speed (5 m/s), layer thickness (100 µm), and scan spacing (150 µm). Only the laser power was varied. Four square plates were printed in each experiment, each one at a different laser energy input, as shown in Table 1. In most cases, a double scanning procedure was used to improve consolidation. At the end of the build, the anchor film was easily detachable from the plates.

In Table 1, in the column named “Print completion”, n/a means “not achieved” because the LS machine aborted due to curl and dislodgement of the part. The numbers in the “print completion” column mean the number of the square plates designed over the thin anchor film that could be laser sintered. In the columns for “Laser power”, where two laser powers are shown, for example, 25 + 40, it means the sintering area of the plate was scanned twice, first with 25 W and a second time with 40 W of laser power.

### 2.3. LS of PA12 at Lowered Bed Temperature

PA12 can be printed in any LS or HSS machine as its optimum bed temperature is <200 °C; however, to demonstrate the applicability of the film anchor method to other polymer materials, PA12 square plates were also laser sintered at lower temperatures compared with the standard conditions usually used for PA12. The anchor film was used to control effectively curling under these low-temperature conditions. The process settings are shown in Table 2. The standard or optimum bed temperature (that is, without the support film) for PA12 is ~167 °C. A single scan was sufficient for PA12 even when the build-bed temperature was lowered below the standard one (167 °C).

### 2.4. Characterization Methods for the Laser-Sintered Parts

The straightness of the laser-sintered plates’ edges, and the sharpness of their corners, are a good first indicator of the quality of the printing. To provide an overview of the edge of the plates and observe the curling effect, a Keyence Digital Microscope VHX-5000 (Keyence, Osaka, Japan) was used.

Next, the densities of the printed parts were measured with a Kern ACS 220-4 density meter (KERN, Balingen, Germany), according to ISO 1183-1:2019 [25] norm. The density indicates whether the sintering quality in terms of consolidation decreases to an unacceptable level, which would lead to a large drop in strength.

A JEOL JSM 7800-F Field Emission Scanning Electron Microscopy (FE-SEM) (JEOL, Tokyo, Japan) at an operating voltage of 5 kV was used to examine the void content of the sintered plates of PET and PA12 when the bed temperature was lowered. The sintered plates were first cryogenically fractured to examine their cross-sectional morphology. The SEM’s low electron detector (LED), (JEOL, Tokyo, Japan) was used to obtain a high-resolution overview of the void content for the plates printed at different bed temperatures. Sputter-coating with gold-palladium was first applied to all the samples to reduce the surface charging during SEM imaging.

## 3. Results and Discussion

**Figure 2:**
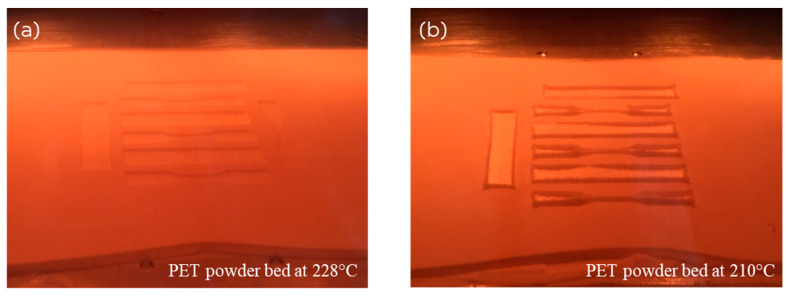
(**a**) Printing with the PET powder bed at 228 °C (optimum conditions for printing without curl) and (**b**) Printing with the PET powder bed at 210 °C (curl is obstructive now). Rectangular bars have dimensions in accordance with ASTM D790 [26] (length of 64 mm × width of 12.7 mm × thickness of 3.2 mm), and tensile bars have dimensions in accordance with ASTM D638 Type V [27].

Figure 2 shows the difference between the LS of PET bars at (a) the optimal bed temperature of 228 °C and (b) the lower bed temperature of 210 °C, both without the anchor support. It is obvious that the curl becomes too severe at 210 °C (Figure 2b). The bars protruded above the powder bed, causing them to be displaced by the powder re-coater, which led to the print being aborted. Consequently, the attempt to laser sinter PET without a support film and at a bed temperature of 210 °C failed due to excessive curling. The operation was not possible at all.

### 3.1. Low-Temperature LS of PET with a Thin Anchor Film

**Figure 3:**
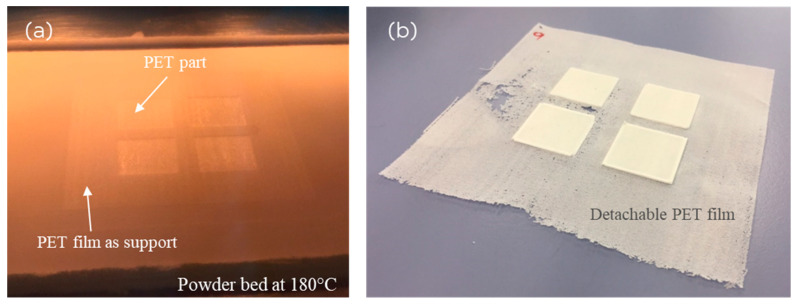
(**a**) Printing of four PET plates with a low bed temperature of 180 °C; (**a**) ~70 μm thick anchor film was printed, and the plates were printed over it. (**b**) After extraction from the print bed, four PET plates with the anchor film attached. The film was printed under conditions where it is weak and brittle and can be detached easily without damaging the articles. Each of the 4 square plates is 30 mm × 30 mm.

Figure 3 displays the method proposed here for printing with a low bed temperature. A thin PET film was printed at the start. A low laser power (16 W) was chosen to create a relatively porous and weak film, allowing it to be easily broken off later to extract the printed parts. The film was printed with an overhang, spanning an area larger than the built objects, consisting of four square plates. The purpose of the film was to serve as an anchor. When the article was printed over it, the curl and retraction were prevented by the friction of the film on the powder bed. As shown in Figure 3b and the summary of the laser-sintered parts in Table 1, with the support of the thin PET anchor film, LS of PET powders at lower temperatures became possible when using a few optimized sintering conditions. Unlike the literature processes using 13 mm thick support plates bolted to the machine [18], here, an in situ printed thin film was sufficient to prevent curl. This breakaway film was easy to remove due to its thin structure (Figure 3b).

As shown in Table 1, the PET parts were laser sintered with both variable powers and a number of scans at different lower temperatures to achieve a systematic study and better understanding of the low-temperature printing window. In comparison with Exp. A1, Exp. A2 used a lower build-bed temperature of 200 °C. By printing first an anchor film at 200 °C, Exp. A2 suggested that it is possible to print plates with relatively good aesthetics when the plates were printed over the anchor film with a single scan at 35 or 45 W. In Exp. A3, a build-bed temperature of 200 °C was used, and again, a base anchor film was printed first, and then the parts were printed over it but with double scans. With scans of 35 W + 35 W and 40 W + 40 W, it was also possible to print parts with relatively good aesthetics. The bed temperature was lowered further to 190 °C (Exp. A4), 180 °C (Exp. A5), and 170 °C (Exp. A6). With proper optimization of the laser power of the base film and printed parts, it was always possible to achieve successfully laser-sintered parts with precise dimension control. However, in Exp. A7 and Exp. A8, when the bed temperature was further lowered to 160 °C and 150 °C (80 °C lower than the standard printing temperature), the build could still be completed, but the parts showed strong curvature after sintering.

Upon completion of the sintering process, the support films of the samples in Exp. A2–A8 were easily peeled off without damaging the plates. Thus, the results suggest that with this method, there is a potential to use PET with commercial LS and HSS machines where the bed temperature is limited to a maximum of 200 °C.

**Figure 4:**
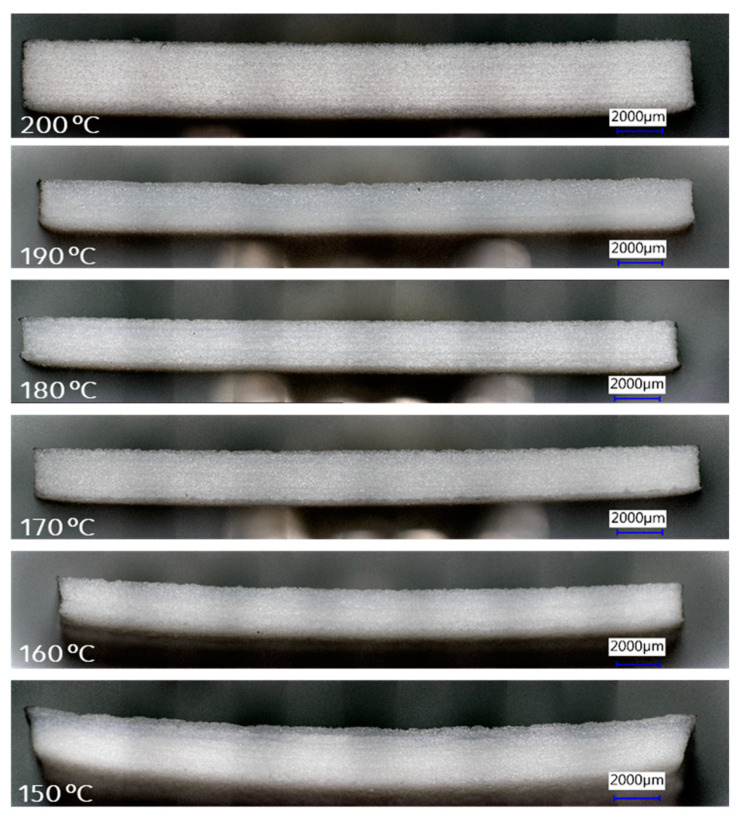
Keyence digital microscopy images of the side view of printed PET parts at different build-bed temperatures as indicated in the images with experimental conditions (A3, A4, A5, A6, A7, and A8) described in Table 1. The part printed at 200 °C (Exp. A3) is thicker than other parts because it was printed with more layers during that specific experiment. The banding is an optical artifact arising from overhead lighting.

To visualize the curvature of the plates, pictures of the edge view were taken and are shown in Figure 4 (Table 1 Exp. A3–A8). It is obvious that parts laser sintered at 170–200 °C have the least curvature. For parts printed at 160 °C and 150 °C, there is a clear curvature observed from the edge view of the printed plates after completing the sintering process.

Note that the plate printed at 200 °C (Figure 4) is thicker since more printing layers were used in the first set of samples for printing. It was observed that print stoppage at lower temperatures typically started from the curl in the first few layers during printing. If the curl is controlled in the first few layers, the printing can continue, and adding more layers does not influence the degree of deformation and cause machine stoppage. Since our interest was in printability at lower bed temperatures, we reduced the number of layers afterward for the efficiency of the experiments (reduced build times). Hence, the plates sintered at 190 °C and lower were made with fewer but constant number of layers, so they appear thinner in Figure 4 than the plate sintered at 200 °C. The curl between samples can be compared despite the difference in thickness of the plate made at 200°C.

The terms “curl” and “warpage” are sometimes used loosely and synonymously [5]. However, Schmid distinguishes [28] two types of part deformation during LS: curl and warpage. Curl occurs in the build phase, while warpage occurs in the post-build when parts are left to cool down in the bed before retrieval. Schmid explained the difference between curl and warpage. In curl, the thickness at the center of the plate is correct, but at the edges, it tapers to a lower thickness, i.e., there is a “loss of material” in the part. The LS process is far from isothermal: the powder in the bed is at a fixed temperature ~5–25 °C below T_m_ in a standard process; the powder is heated above the T_m_ in the desired area to fuse it; then a new layer of powder is added which is lower in temperature than the bed, causing cooling of the melt below, inducing crystallization and shrinkage. In LS at low temperatures, the difference with T_m_ increases, and curling primarily occurs due to increased thermal gradients. This uneven heating and cooling lead to anisotropic shrinkage, where the part contracts differently along various directions due to directional cooling. In semi-crystalline polymers, this effect is amplified by rapid crystallization, which introduces additional shrinkage and residual stress as crystalline regions occupy less volume than amorphous ones.

“Warpage” refers to parts that have been built properly with uniform thickness along the length, but which severely twisted during the cooling period after completing the sintering process. In practice, deformed parts might also arise from a combination of curl and warpage [9]. Since the thickness at the corners of the plates in Figure 4 is substantially the same as at the center, the observed curvature in parts printed between 150 and 160 °C probably arose from a post-build warpage rather than curl. If the curling during sintering had been severe, the machine would have stopped as the part would have been dislodged from its position in the bed. In the current case, the LS process was completed successfully; however, the warpage exhibited in the parts printed at temperatures between 150 and 160 °C indicates that the temperature gradient and cooling time after sintering could be further optimized to minimize warpage.

The densities of the PET plates from different temperature conditions were measured and are shown in Table 3. Compared with the density achieved from the standard printing procedure for PET (1.36 g/cm^3^. ref. [18]), the densities of the parts printed in lower build temperature conditions showed a slight decrease (~2.0%) until the bed temperature was below 170 °C. A decrease in density (~3.7%) was found for the part printed at 160 °C. A more prominent decrease in density, however, was found for the part printed at 150 °C (~11.8%). The density of the parts indicates the void content, and since tensile strength is correlated with both void content and density, these results provide important insights. The density results suggest that the mechanical strength of PET parts printed with the lowest bed temperatures, such as 160 °C or 150 °C, would be significantly lower than those printed with optimum conditions; however, parts laser-sintered at bed temperatures between 170 °C and 200 °C could still achieve acceptable mechanical strength, with the density result from 190 °C being closest to the standard.

### 3.2. Cross-Sectional Morphology of the Laser-Sintered PET Parts

**Figure 5:**
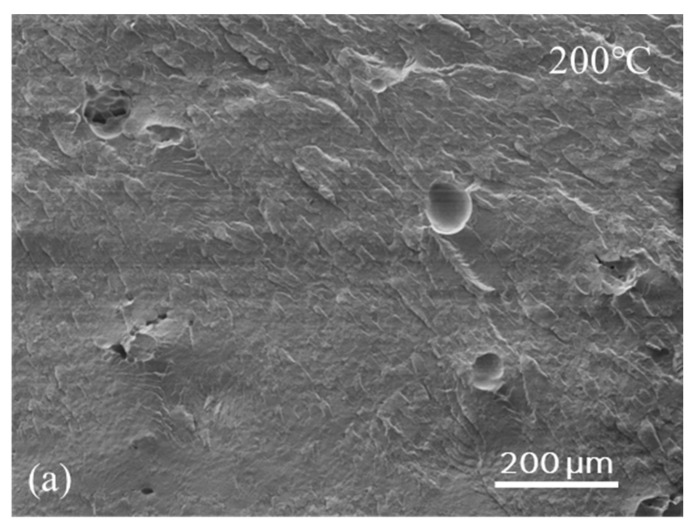

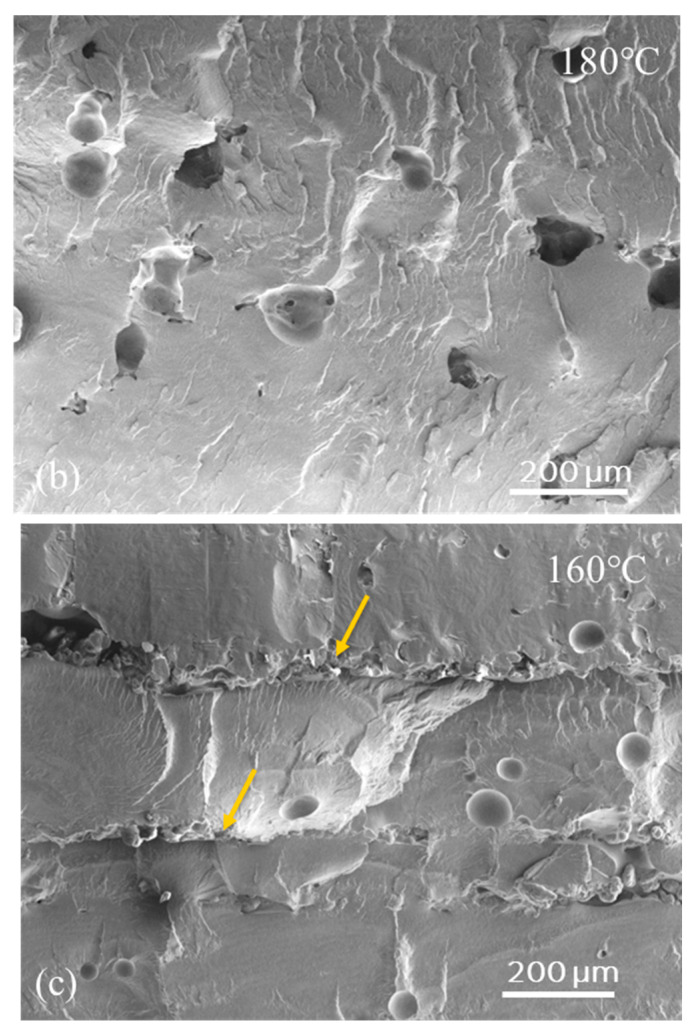
SEM images of the fractured cross-section of printed PET parts from (**a**) build-bed temperature 200 °C, (**b**) build-bed temperature 180 °C, and (**c**) build-bed temperature 160 °C: a layered structure indicated by yellow arrows is visible.

Unlike injection molding, the LS process leaves voids and sometimes layered or stratified structures in the cross-section, and this affects the quality of the internal consolidation, which in turn affects the tensile strength and elongation-to-break. With the best semi-crystalline polymers for LS, such as PA12, PA11, and PET, 2–5 volume % residual voids can be expected even under the best optimized LS conditions [29]. With other polymers, the void content can be as high as 50–60% [29]. A question to investigate in this study was whether using a low bed temperature would increase the voids and compromise the quality of internal consolidation to the point where the parts become unusable. Hence, fractured cross-sections of the parts described in Table 1 were examined by SEM. A few representative images of these sections are shown in Figure 5 (200 °C, 180 °C and 160 °C). These images clearly show that a comparable state of consolidation was achieved for PET parts printed at both 200 °C and 180 °C without visible layer structures. This level of consolidation is similar to that of parts produced under standard PET LS conditions, using bed temperatures of 228–233 °C. Based on the image analysis on the SEM image of the PET cross-section morphology at 200 °C, the void content was similar to that produced under the optimal condition with a void percentage of 1.5 ± 0.2 vol.% [12]. A slight increase in the amount of void content to ~1.8 ± 0.2 vol.% was observed when the PET plates were printed at 180 °C. The results suggest that even when sintering PET using bed temperatures as low as 180 °C, the PET was still above the softening temperature for a sufficiently long time. Therefore, there were no layered structures visible. However, layered structures from the LS plates became visible when the PET was printed with a bed temperature of 160 °C (Figure 5), even with the anchor film. Many laser-sintered polymers which are under-consolidated exhibit a layered structure in their cross-sections, resembling sedimentary rock. A layered structure generally results in reduced interlayer strength, leading to lower tensile strength in the z-direction, and potentially causing delamination. This means if using a bed temperature of 160 °C for PET, the article would show a higher anisotropic mechanical strength, compared with parts printed under standard conditions for PET.

This work suggests it is feasible to use the double scan and an in situ printed thin anchor film to produce PET parts of a similar density and non-layered structure at bed temperatures of 170–200 °C, comparable to those achieved under standard conditions (bed temperature of 228–233 °C). Therefore, a commercial LS machine limited to a maximum operating bed temperature of 200 °C may be usable for LS PET. In this process, the frictional force generated by moving the thin film over the underlying particulate powder layer restrains the sintered parts from shrinking and curling. The powder particles under the film are semi-sintered to the film, which acts as a further restraint to prevent movement of the film, and the weight of additional layers of powders increases the frictional force needed for sliding the film (from the shrinkage from significant thermal gradients and crystallization). In this way, an in situ laser-sintered thin film, which is easily separable, is effective in preventing part curling during low-temperature printing.

For PET, we did not go to a single scan with very high laser powers to compensate for the lowered bed temperatures. Excessively high laser power (high energy density) can result in burning and smoke generation [30]. Instead, we used a double scan for the PET, which admittedly compromises the production speed. At this stage, the desire was to show that PET powder can be laser sintered in commonly available LS machines using bed temperatures limited to a maximum of 200 °C, without machine stoppage, without part-distortion, and drastic decrease in part density. Exploration needs to be conducted to achieve part production of PET at lower bed temperatures with the support film and a single scan without smoke generation and without compromising the density.

### 3.3. Low-Temperature LS of PA12

To study the general applicability of the process for polymers other than PET, PA12 was laser sintered with bed temperatures lower than the standard. Typically, PA12 needs a bed temperature of ~167 °C, and thus, it is not a challenge to sinter it in all available LS and HSS machines. However, PA12 suffers from a fast rise in molecular weight in powder exposed to 167 °C for hours during a build [16], thus limiting its reuse. The rise in molecular weight is around 1.7× in the first hour at 167 °C [11]. This rise means the melt viscosity significantly increases, and hence, reuse of the unsintered powder in a subsequent build leads to printing defects such as orange peel [17]. To overcome this, 30–50% virgin powder needs to be added to the heat-exposed powder. Thus, a lower build temperature for printing PA12 would be very beneficial from the perspective of preserving unsintered but heat-exposed powder.

Table 2, Exp. B1 shows the effect of lowering the build-bed temperature from 167 °C to 160 °C (without the support film). Due to curl, it was already impossible to complete the printing of the square plates at any laser-power setting. Exp. B2 in Table 2 illustrates the impact of first printing an in situ PA12 film layer, followed by the plates, with a build temperature of 160 °C. Unlike Exp. B1, the curl was eliminated, and the square plate could be sintered satisfactorily with all four laser powers using a single scan. Exp. B3 and B4 show the effect of LS with an in situ film layer and then sintering the plates over it, with the build temperature lowered to 155 °C and 150 °C, respectively. Still, the curl was controlled, and plates 2–4 were satisfactory. Exp. B5 shows the effect of LS with the build temperature lowered to 145 °C. No machine-stopping curl was observed and the square plate could be laser sintered satisfactorily for power settings 3 and 4. Exp. B6 shows that with the build temperature lowered to 140 °C, the curl was acceptable for completing the printing at power settings 3 and 4, but the part warped during cooling after printing. When the build-bed temperature decreased to 130 °C, it was impossible to complete the printing of the square plates at any power setting.

In general, as the build-bed temperature was lowered and the difference between bed temperature and T_m_ increased, higher laser powers were needed (see Table 2). However, because the difference between the lowered bed temperature and the T_m_ was relatively smaller for PA12 (at 140 °C, it is 27 °C below the standard 167 °C) than for PET (78 °C below optimum if the bed temperature is 150 °C, compared with the optimum of 228 °C), a different trend can be observed. Table 2 shows that the PA12 part could be laser sintered with a single scan, whereas in most of the low-temperature printing with PET, double scans were required (see Table 1).

**Figure 6:**
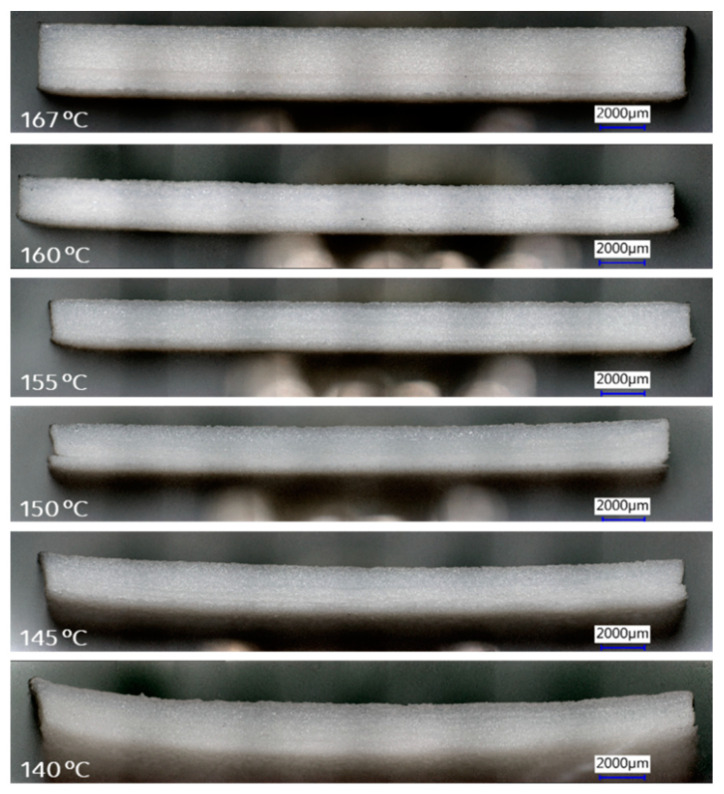
Keyence digital microscopy images of side view of printed PA12 parts at different build-bed temperatures. The part printed at 167 °C is thicker than other parts because it was printed with more layers during that specific experiment. The banding is an optical artifact arising from overhead lighting.

Figure 6 shows the edge views of the PA12 parts printed at lower temperatures with the in situ printed anchor film. Note again that the part printed at 167 °C is thicker because more layers were printed. No clear curl was present at 160 °C and 155 °C. Even at 145 °C and 140 °C, the plates were printable with slight bending of the printed parts. However, the thickness at both ends of the parts is similar to the thickness of the part’s center. As discussed for PET, the observed bending curvature at 145 °C and 140 °C is likely to be due to the post-printing warpage rather than curl [28].

The densities of the PA12 plates made under different bed temperatures were measured and are shown in Table 4. Compared with the density achieved from the standard LS procedure for PA12 (0.99 g/cm^3^), the densities of the parts printed in lower build temperature conditions are similar or even slightly increased to 1.00 and 1.01 g/cm^3^. The density of injection-molded PA12, according to data published in various suppliers’ data sheets, is in the range of 1.01–1.03 g/cm^3^. The results here suggest that the level of consolidation for the PA12 parts printed at lower temperatures is comparable to, or even better than, that achieved at the standard bed temperature of 167 °C and is also quite close to the injection-molded value (1.03 g/cm^3^).

**Figure 7:**
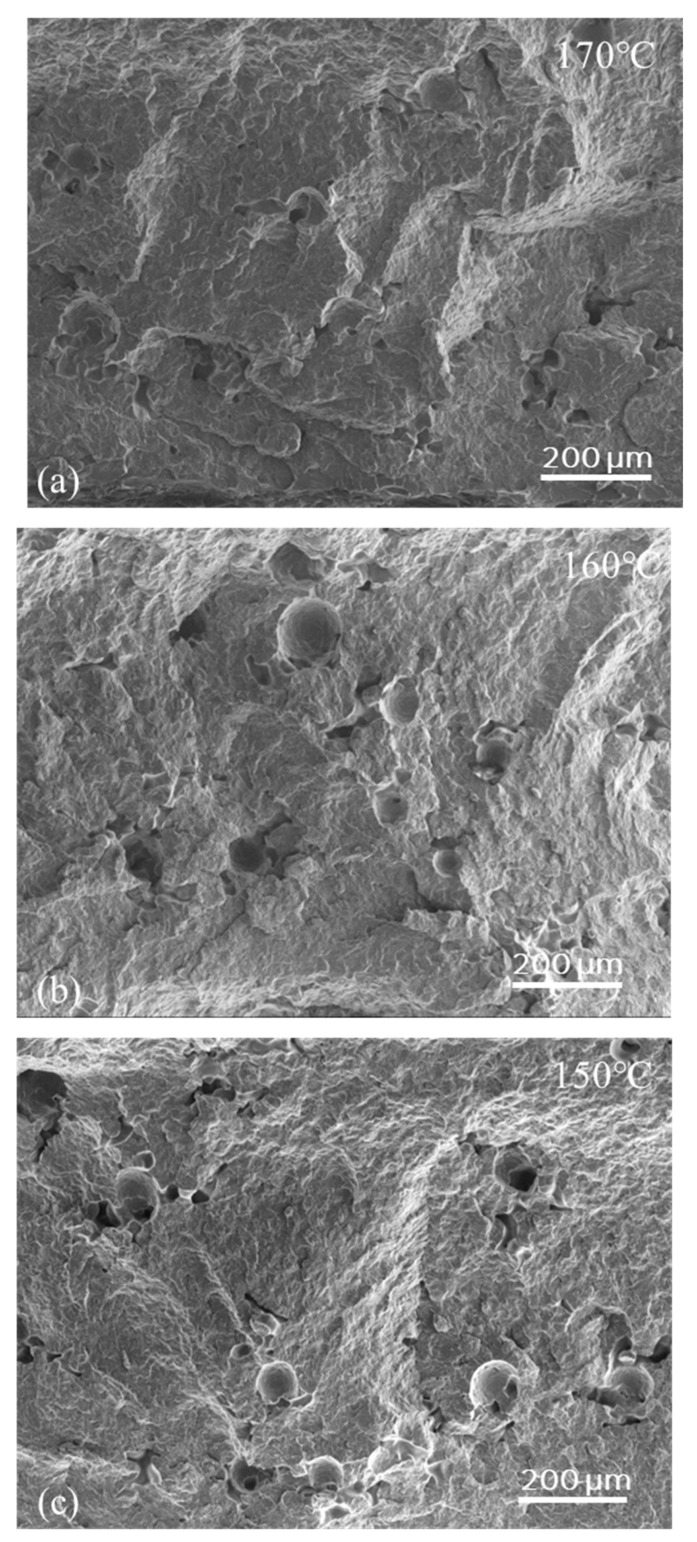
SEM images of the fractured cross-section of printed PA12 parts from (**a**) build-bed temperature 167 °C, (**b**) build-bed temperature 160 °C, and (**c**) build-bed temperature 150 °C. There is no layered structure visible.

The SEM was used to examine the cross-section of the PA12 bars printed with standard bed temperature of 167 °C and lower temperatures of 160 °C and 150 °C, respectively. As shown in Figure 7, a high consolidation level without any layered structures was present in all three sintered plates. This finding is consistent with the similar density for the PA12 parts sintered at different temperatures. The bar printed at the standard temperature of 167 °C shows a slightly lower porosity (Figure 7a), but the percentage of porosity for all three printed samples (Figure 7a–c) was generally ~2 vol.%, which is in the range of the porosity reported in the literature for PA12 [29]. The porosity estimated from the SEM analysis aligns with the expected density values, showing 0.99 to 1.01 g/cm³ for laser-sintered PA12 (as shown in Table 4) compared to 1.03 g/cm³ for fully consolidated injection-molded PA12.

Here, we demonstrated a successful printing of PA12 parts without any density decrease when the bed temperature was up to 27 °C lower (up to 17 °C if curvature is included) than the standard printing process. Considering the degradation of PA12 powder in the bed, even a 17–27 °C decrease in bed temperature is useful, as the chain growth reaction rate will be reduced [16]. Low-temperature printing with the in situ printed thin anchor film could likely be further improved by optimizing the laser energy density, potentially enabling this method to be extended for PA12 at bed temperatures below 140 °C.

To determine further whether this method of LS with lower bed temperatures would allow the production of relatively complex shapes, a chainmail was laser sintered from PA12 with a bed temperature of 150 °C, which is 17 °C below the standard bed temperature for PA12. 

**Figure 8:**
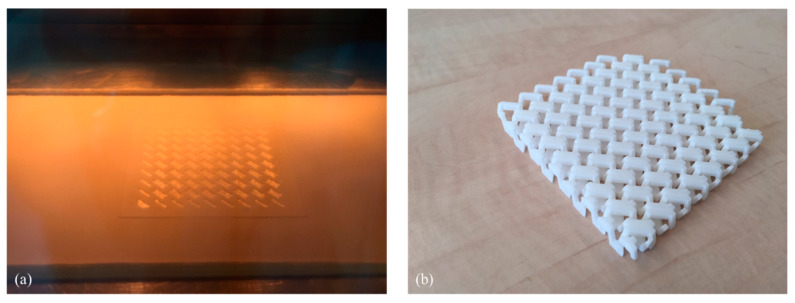
(**a**) Printing a chainmail from PA12 at a low build-bed temperature of 150 °C (standard is 167 °C). (**b**) The complex printed chainmail with dimension of 100 mm × 100 mm × 12 mm.

3D-printed chainmail combines flexibility and rigidity in the article. The linking elements can be octahedrons, pyramids, or rhomboids. Rhomboids were chosen for this demonstration. Normally, for PA12 with a bed temperature of 150 °C, the curl during printing would be so severe that the machine would abort the print. With our low-temperature printing method, a 110 mm × 110 mm PA12 anchor film was printed first. Figure 8a shows the print bed at 150 °C during the printing of the chainmail over the anchor film, where it was evident that the process could proceed without interruption from curling. Figure 8b shows the chainmail after detachment of the anchor film and de-dusting. There was no curl or warpage. The laser power can be further optimized to improve the parts, but this clearly demonstrates the feasibility of complex-shape realization using lowered bed temperatures with even PA 12.

For PA12, there is no limitation as such in the machine to attain the optimum bed temperature. However, the results suggest that LS at a lower build-bed temperature benefits the user not only in energy saving but also in reducing powder waste via controlling the molecular weight rise [17] in the unsintered but heat-exposed powder left after a build. These features lead to environmental benefits as well as cost reduction. The method may be extendable to PEEK as well, where severe powder degradation and machine availability are problems.

### 3.4. Flexible Versus Rigid Supports for Low Bed Temperature LS

The results from LS of PET and PA12 with the lower bed temperatures using a flexible anchor to control curl will be compared with previous attempts at low-temperature printing using a rigid support. The compromises of various methods will be assessed.

It is worth highlighting the ease of fabricating and removing the flexible support in this paper compared with previous rigid support methods used to overcome curl and warpage during low bed-temperature LS. In Menge and Schmid’s method [20], if the printing powder is PA12, a building platform (the rigid support) had to be engineered out of a composite material made of carbon-fiber-reinforced PA12 bonded to the “adjustment platform” (part of the machine). Though the thickness of the building platform, the method used for its fabrication (injection molding or extrusion), and the ease of debonding the parts from the building platform were not revealed, it can be expected to be much more complicated than the in situ printed anchor film method described here. In the method for LS of PEEK {20] at low bed temperature, a metal grid needed to print a thick PEEK sheet that was flat, and the article has to be printed on breakable struts which would be cumbersome to design and print. In the work of Niino et al. [18,19], the building platform (made of the same polymer as the printing powder) was 13 mm thick and was bolted to the machine. In these methods, external fabrication of the building platform and attachment to the machine is needed. The ease of detachment of the parts from the base plate was not disclosed. Therefore, for a different printing powder, for example, PET or polypropylene, a different rigid plate would have to be built and attached to the machine. Here, the building platform is an in situ printed film generated from the material being laser sintered, and no attachment to the machine is needed by adhesives or bolting [18,19]. Second, regarding the LS of PA12, with rigid support methods as reported by Menge and Schmid [20], they printed with bed temperatures between 80 and 120 °C (that is lower than what could be achieved here with PA 12). However, the density of their printed parts decreased to 0.886–0.9 g/cm^3^ versus 0.99 for the standard process. The lowest bed temperature we could achieve for PA12 LS without machine stoppage with the in situ film method was 140 °C (lower the bed temperature by 27 °C), and printed PA12 parts showing a density of ~1.01 g/cm^3^, which is higher than the standard process and close to the density value of injection-molded PA12, suggesting minor loss in mechanical strength will be incurred. In addition, we printed a complex article with bed temperature lowered to 150 °C.

## 4. Conclusions

This work aimed to achieve the LS of PET, with a melting point of 240–250 °C, in “standard machines” that are limited to a maximum powder-bed temperature of 200 °C.

The main difficulty was how to counter-act curl, which causes part-distortion, and results in machine stoppage if too severe. Curl increases when the bed temperature is >5–25 °C below the T_m_ of the semi-crystalline polymer. To prevent curl and part-distortion, a support layer was used as a base for printing the article. Unlike previous studies on LS at low bed temperatures, which used a thick (~13 mm), rigid support, this approach employed an in situ printed monolayer PET film. This thin film restrained the printed part’s shrinkage through frictional resistance and was easily removable at the end of the build process.

With the use of the anchor film, PET parts were successfully printed without machine stoppage at bed temperatures as low as 150 °C, which is about 80 °C lower than the bed temperature for a regular process for PET without the anchor film. With the bed at 200 °C, we could print PET with a single scan (no decrease in productivity) and attain a density of 1.34 g/cm^3^ (a decrease of 1.5%). For bed temperatures of 190 °C and below, we had to use a double laser scan; hence, the printing speed was reduced by 50%. The densities of the PET parts printed with lower build temperatures (190–160 °C) showed a slight decrease of ~2.0%, but the density decreased significantly when the bed temperature was reduced to 150 °C.

The proposed method was not restricted to PET, it was shown to work also with PA12, the most commonly used LS polymer powder. All LS and HSS machines can print PA12, as its optimum bed temperature is ~167 °C. However, lower bed temperatures reduce energy consumption and decrease the degradation rate of PA12. Interestingly, the density of the PA 12 part printed at a bed temperature of 150 °C was higher than that achieved at the “standard” bed temperature of 167 °C. A complex part was also successfully printed with PA12 using a bed temperature of 150 °C.

It is anticipated that this method of low bed-temperature LS can also be used for HSS and would be useful for higher-temperature polymers like PET.

## Figures and Tables

**Table 1:** Printing of PET plates with anchor film and low build-bed temperatures (that is, below the optimum for PET) with different experimental conditions (A1 to A8). n/a = not achieved.

Exp. nr	Building-Area Temperature (°C)	Laser Power					Print Completion
		Anchor Film (Watts)	Part 1 (Watts)	Part 2 (Watts)	Part 3 (Watts)	Part 4 (Watts)	
A1	200	0	25 + 25	30 + 30	35 + 35	40 + 40	n/a
A2	200	16.5	25	30	35	40	3, 4
A3	200	16.5	25 + 25	30 + 30	35 + 35	40 + 40	3, 4
A4	190	16.5	25 + 40	30 + 40	35 + 40	40 + 40	3, 4
A5	180	25	45 + 45	30 + 45	35 + 45	40 + 45	1, 4
A6	170	25	45 + 45	30 + 45	35 + 45	40 + 45	1, 4
A7	160	25	45 + 45	30 + 45	35 + 45	40 + 45	Curved
A8	150	25	45 + 45	30 + 45	35 + 45	40 + 45	Curved

**Table 2:** Lowering build-bed temperature for PA12 powder below the standard (167 °C), with and without anchoring film and different experimental conditions (B1 to B7). n/a = not achieved.

Exp. nr	Building-Area Temperature (°C)	Laser Power					Print Completion
		Anchor Film (Watts)	Part 1 (Watts)	Part 2 (Watts)	Part 3 (Watts)	Part 4 (Watts)	
B1	160	0	17	20	23	26	n/a
B2	160	15	17	20	23	26	1, 2, 3, 4
B3	155	15	17	20	23	26	2, 3, 4
B4	150	15	17	20	23	26	2, 3, 4
B5	145	15	17	20	23	26	3, 4
B6	140	17	17	20	23	26	3, 4
B7	130	17	20	23	26	29	n/a

**Table 3:** Density of printed PET parts at different build temperature conditions. Under standard conditions, an anchor film is not needed. With the lower bed temperatures, an anchor film is needed.

Bed Temperature (°C)	228 (Standard)	200	190	180	170	160	150
Density (g/cm^3^)	1.36 ± 0.02	1.34 ± 0.02	1.35 ± 0.02	1.33 ± 0.02	1.33 ± 0.02	1.31 ± 0.02	1.2 ± 0.01

**Table 4:** Density of printed PA12 parts at different build temperature conditions.

Build Temperature (°C)	167 (Standard)	160	155	150	145	140
Density (g/cm^3^)	0.99 ± 0.01	0.99 ± 0.01	1.00 ± 0.01	1.01 ± 0.01	1.00 ± 0.01	1.00 ± 0.01

## Data Availability

Data are contained within the article.
